# Optimization of the Elastic Modulus of the Filler in High-Rib Thin-Web Grid-Stiffened Panels with Bending Forming Process

**DOI:** 10.3390/ma18163782

**Published:** 2025-08-12

**Authors:** Siyu Nan, Xinlong Zhang

**Affiliations:** College of Mechanical and Electrical Engineering, Northeast Forestry University, Harbin 150040, China; nansiyv@163.com

**Keywords:** aerospace, grid-stiffened panel, roll-bending forming, high-rib thin-web, filler elastic modulus

## Abstract

Grid-stiffened panels are indispensable in aerospace applications, valued for their lightweight nature, high strength, and excellent deformation resistance. However, the roll-bending forming process of these panels is plagued by critical defects such as rib buckling and uneven skin deformation, which undermine structural quality and performance. Fillers are commonly employed to mitigate these issues, yet there remains a lack of systematic guidance for optimizing filler parameters—particularly the elastic modulus—that is tailored to high-rib thin-web configurations. This study focuses on high-rib thin-web grid-stiffened panels, aiming to address this gap by exploring the optimization of filler elastic modulus. By delving into this critical parameter, the research seeks to lay the groundwork for enhancing forming precision in roll bending, offering valuable insights for advancing high-quality manufacturing of aerospace components.

## 1. Introduction

In modern aerospace engineering, saving even a few kilograms of structural mass can translate into millions of dollars in fuel costs and dramatically improved mission performance [1,2]. Grid-stiffened panels are skins reinforced by an array of intersecting ribs and can significantly reduce structural weight while maintaining high strength and resistance to deformation [3]. By carefully optimizing rib layout and dimensions, these panels can achieve the same load-bearing capacity as a solid plate at a fraction of the weight, while still resisting deformation under extreme service conditions. As a result, integral grid-stiffened panels produced via one-piece molding have seen widespread adoption in critical aerospace components such as aircraft wings, launch vehicle propellant tanks, and rocket casings [4,5,6].

The bending forming methods of the integral panel mainly include shot peening forming, incremental bending forming, creep aging forming, electromagnetic forming, and roll-bending forming [7]. Among these, pure mechanical bending forming processes shape the material directly by mechanical force without requiring thermal energy, chemical media, or high-energy equipment. These processes are stable and controllable, cost-effective, impose low requirements on the material’s thermal sensitivity, yield good surface quality, and are suitable for large-scale production. As a result, roll-bending forming and press-bending forming have become mainstream, focusing on the forming research of metal integral panels.

In the manufacturing and forming of grid-stiffened panels, curvature radius and generatrix straightness are the most critical technical parameters, directly determining panel quality and performance and playing a decisive role in subsequent applications. However, during production, defects such as stiffener buckling [8,9], local skin indentation, or even fracture can occur, adversely affecting curvature radius and generatrix straightness, reducing structural strength, and greatly diminishing forming accuracy.

Numerous researchers have examined the bending behavior of grid-stiffened panels from various perspectives. Zhou et al. [10,11,12] focused on the buckling behavior of stiffened plates under bending loads, examining the influence of various factors on their buckling strength and coefficients. Rahbar-Ranji et al. [13] analyzed how plate, torsional, and web buckling modes interact and couple in flat-bar stiffeners under varied loading and boundary conditions. Chagraoui et al. [14] introduced curved-grid stiffening for T-stiffened composites and used a genetic algorithm to refine rib morphology and layout. Liu et al. [15], using finite element methods and experimental techniques, compared filling roll bending and incremental bending forming processes for integral panels and investigated their applicability.

To mitigate these defects, researchers have introduced fillers into the roll-bending process. Zhang et al. [16] were the first to apply filling roll-bending technology to integral panels and carried out experimental studies demonstrating that rubber or plastic can be used as filler to form integral panels during roll-bending forming. Liu et al. [15] and Xiao et al. [6] established the finite element model of the integral panel filling roll bending and analyzed the filler’s influence on equivalent stress, shear stress, and equivalent strain. They propose that the principle of this process is to improve the load-bearing behavior of the ribs and enhance their resistance to instability by adding filler. Yin et al. [17] compared the effectiveness of rubber and polyethylene as fillers, evaluating their impacts on panel forming performance. Li et al. [18] investigated the factors influencing discrete-filling-assisted roll-bending forming of integral panels. Li et al. [19] explored the influence of filler parameters on panel curvature radius, the neutral layer, and surface quality. It has been found that filling-assisted forming can effectively improve load distribution in panels, protect the ribs, and enhance forming performance.

However, current research has yet to provide a systematic elucidation of the filling-assisted mechanism or clear guidelines for optimizing filler parameters, resulting in existing research findings being unable to provide effective theoretical support for the practical production of high-rib thin-web grid-stiffened panels. To address this research gap, this study, grounded in rib-buckling theory, proposes a calculation method for the filler’s elastic modulus specifically tailored to high-rib thin-web grid-stiffened panels. Matching the filler stiffness with the ribs’ critical buckling load allows determination of the optimal elastic modulus that synchronizes deformation between ribs and filler. Subsequently, simulations evaluated stress and strain distributions and forming performance across a range of filler moduli. Ultimately, forming trials on integral aluminum alloy panels validated the theoretical predictions.

## 2. Theoretical Analysis

### 2.1. Analysis and Selection of Plate-Bending Machines

During plate-bending forming, the plate-bending machine plays a crucial role, with the roll arrangement significantly influencing the process characteristics. Based on the number of rollers, plate-bending machines are classified into three-roll and four-roll types. The three-roll plate-bending machine employs an asymmetric roll arrangement, offering advantages such as a simple structure and lower maintenance costs. However, it presents notable process limitations: first, residual straight edges at the ends of the plate are difficult to eliminate completely, leading to geometric defects in the formed parts; second, multiple trial bending iterations are required to approach the target curvature, increasing processing time and limiting the minimum bending diameter.

**Figure 1:**
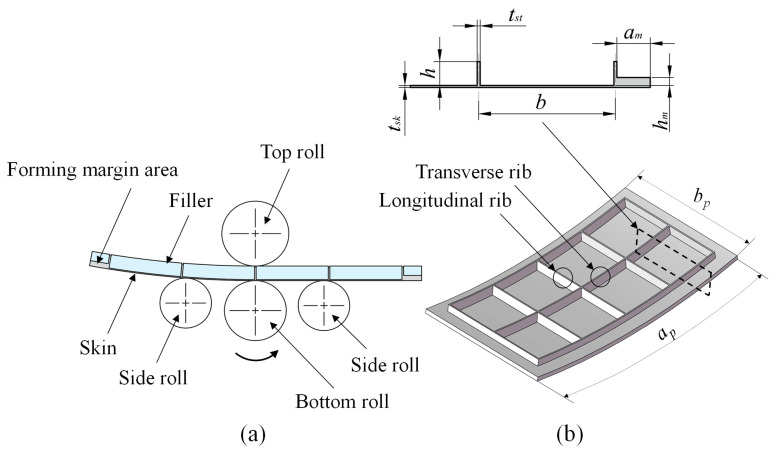
Roll bending model: (**a**) four-roll plate-bending machine model, (**b**) grid-stiffened panel dimensions.

In contrast, the four-roll plate-bending machine uses a symmetric roll arrangement, as shown in Figure 1a, coupled with an active bottom roll displacement compensation mechanism, enabling full-width prebending. This configuration allows for a high forming accuracy of ±0.1 mm, supports a maximum processing thickness of up to 60 mm, and facilitates continuous rolling production, significantly enhancing production efficiency. For roll bending of grid-stiffened panels in the aerospace sector, the four-roll plate-bending machine’s high-precision forming capability ensures dimensional accuracy and geometric conformity to design specifications. Additionally, the clamping action of the top and bottom rolls contributes to panel straightening, improving generatrix straightness. The ability to process ultra-thick plates and support continuous production further positions the four-roll plate-bending machine as the preferred choice for aerospace grid-stiffened panel fabrication.

### 2.2. Theoretical Calculation of Elastic Modulus

Main grid types include upright orthogonal, inclined orthogonal, and equilateral triangular configurations. The upright orthogonal grid features rib-axis distribution aligned with the deformation direction of the panel, which avoids interference from multi-directional deformation components caused by spatial angular deviation between rib orientation and the principal loading direction. In other grid types, this interference forces the computational model to handle mechanical coordination issues in multiple directions simultaneously, thereby greatly increasing computational complexity. Therefore, choosing the upright orthogonal grid for theoretical analysis is more conducive to engineering simplification, and the computational model can be extended to other mainstream grid types.

**Figure 2:**
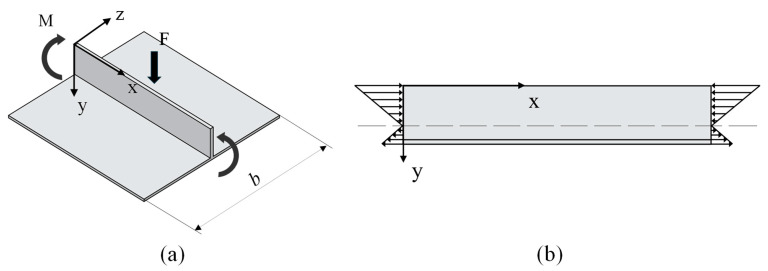
Loading conditions of transverse ribs: (**a**) simplified force bearing model of transverse ribs, (**b**) bending compression combined force bearing model of transverse ribs.

Building on this advantage, this paper analyzes the roll-bending forming of high-rib thin-web grid-stiffened panels with an upright orthogonal configuration as an example. The upright orthogonal grid-stiffened panel model and its specific dimensions are shown in Figure 1b. The calculation method for the filler elastic modulus is based on transverse rib buckling behavior. As shown in Figure 2a, a single transverse rib was chosen as the study subject. Since the rib’s thickness is relatively small compared to its other dimensions, it can be modeled as a vertical thin plate. It should be noted that this thin plate idealization has limitations: first, its accuracy may be compromised near rib intersections due to the development of complex three-dimensional stress states; second, the validity of thin plate theory typically requires the ratio of the plate’s minimum span to its thickness to be greater than or equal to eight, with prediction accuracy decreasing for ribs where this ratio is lower. The primary sources of its deformation are the bending moment and the pressure applied by the top roll of the plate-bending machine. During the process of bending grid-stiffened panels, to protect the forming effect of the ribs, the filler height is usually chosen to be slightly higher than the rib, allowing the top roll to contact the filler and continuously apply pressure, thus providing a bending moment to drive the panel into plastic deformation. Therefore, the loading conditions of the transverse rib can be simplified by considering only the bending moment. Furthermore, due to the presence of the skin, the ribs do not experience pure bending. In this case, the transverse rib can be regarded as a vertical thin plate under combined bending and compression, as shown in Figure 2b, with a certain risk of buckling [10,20].

**Figure 3:**
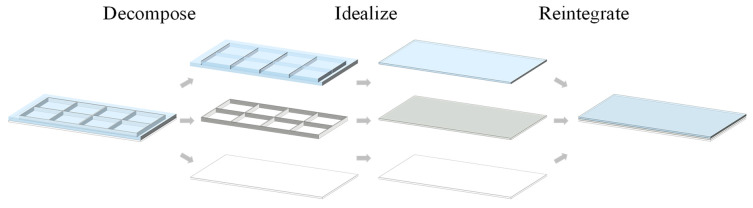
Equivalent modeling procedure of grid-stiffened panels.

Given the complexity associated with analyzing the bending behavior of grid-stiffened panels, a stiffness equivalence method is herein proposed to transform them into an analytically tractable equivalent homogeneous plate model. The detailed procedure is illustrated in Figure 3. First, the grid-stiffened panel is decomposed into three components, namely filler, grid ribs, and skin. Each component is evaluated according to the bending stiffness equivalence principle and then combined to form an equivalent plate. For the grid rib section, the effective bending stiffness of the transverse ribs needs to be calculated based on the equivalent stiffness method for orthogonal grids derived from Timoshenko’s plate and shell theory [21], considering only the contribution of transverse ribs to bending stiffness within the plane of the bending moment. The bending stiffness of a single transverse rib is averaged over the entire panel area due to the distribution of rib spacing, so the effective bending stiffness in the x-direction can be expressed as

(1) $$D_{x}=B/b$$

where $D_{x}$ is the effective bending stiffness in the x direction, B is the bending stiffness of a single rib, and b is the spacing between ribs.

(2) $$B=EI_{1}$$

where E is the elastic modulus of the metal panel, $I_{1}$ is the moment of inertia of the single rib,

(3) $$I_{1}=t_{st}h^{3}/12$$

where $t_{st}$ is the rib thickness, h is the rib height, let

(4) $$D_{x}=EI_{2}$$

(5) $$I_{2}={\;b}_{p}t_{r}^{3}/12$$

$b_{p}$ is the plate width, then the equivalent thickness $t_{r}$ of the grid rib portion can be obtained.

The equivalent thickness of the filler is then calculated. The y–z plane is taken as the section, and $E_{1}I_{1}$ is used to compute the bending stiffness of each filler on that section, including the filler above the forming margin area. $E_{1}$ represents the elastic modulus of the filler, which is assumed by the user, and the total bending stiffness of the filler, $EI_{t}$, is obtained by summing them all.

(6) $$EI_{t}=\sum E_{1}I_{1}$$

Let

(7) $$EI_{t}=EI_{2}$$

(8) $$I_{2}=b_{p}t_{f}^{3}/12$$

$t_{f}$ is the equivalent thickness of the filler.

The calculation method for the forming margin area is the same as for the filler, and the moment of inertia for one side of the forming margin area is

(9) $$I_{1}=a_{m}h_{m}^{3}/12$$

(10) $$EI_{t}=2EI_{1}$$

The equivalent thickness of the forming margin area $t_{m}$ is obtained by combining Equations (7) and (8). By adding the original skin thickness $t_{sk}$ to each equivalent thickness, the total equivalent homogeneous panel thickness $t_{eq}$ is obtained.

(11) $$t_{eq}=t_{r}+t_{f}+t_{m}+t_{sk}$$

This completes the transformation of the grid-stiffened panel into an equivalent homogeneous plate.

**Figure 4:**
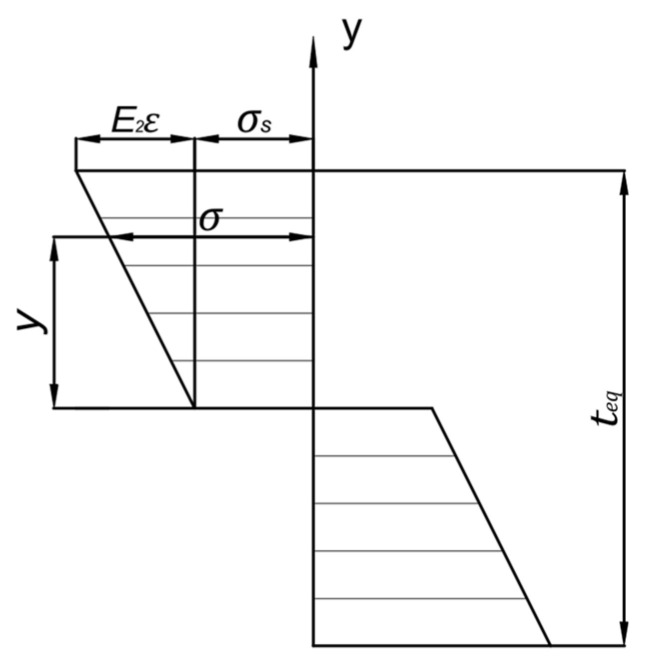
Through-thickness stress distribution in a plate under linear fully plastic bending.

The entire panel undergoes a deformation process that progresses from elastic to elastoplastic, and finally to plastic. When the bending radius is large, the curved panel can be approximated as a straight member, with only tangential stress and deformation taken into account. Under such conditions, the deformation can be treated as linear pure plastic bending. The through-thickness stress distribution of the panel in this linear pure plastic bending state is illustrated in Figure 4.

During the plastic bending process, when the external force is unloaded, the plastic deformation remains, while the elastic deformation part recovers, a phenomenon known as elastic recovery. Assume that $R^{\prime }$ is the curvature radius before springback, and R is the curvature radius after springback, which is also the target curvature radius.

Under the plane assumption, after bending, the cross-section of the sheet remains flat and perpendicular to the neutral axis. From this, the relationship between the linear strain, the distance from the point to the neutral axis, and the curvature can be derived as follows:

(12) $$\varepsilon \;=\;\frac{y}{R^{\prime }}$$

In linear pure plastic bending, the stress value at any point along the plate thickness direction can be expressed as

(13) $$\sigma =\sigma_{s}+E_{2}\varepsilon$$

σ is the workpiece stress, $\sigma_{s}$ is the initial yield stress, and $E_{2}$ is the material’s linear hardening modulus.

The relative curvature radius $r^{\prime }$ before springback can be expressed as

(14) $$r^{\prime }=\frac{R^{\prime }}{t_{eq}}$$

The relative curvature radius r after springback is denoted as

(15) $$r=\frac{R}{t_{eq}}$$

The change in the bending moment is expressed using the relative bending moment m:

(16) $$m\;=\;\frac{M}{W\sigma_{s}}\;={\;K}_{1}\;+\;\frac{K_{0}}{2r^{\prime }}$$

M is the bending moment on the section, W is the section modulus, $K_{1}$ is the shape factor, for a rectangular section, take $\;K_{1}=\;1.5$, $K_{0}$ is the material’s relative hardening modulus.

The relationship between r and $r^{\prime }$ satisfies [22]

(17) $$r^{\prime }=\frac{r}{1+2m\frac{\sigma_{s}}{E}r}$$

The corrected curvature radius can be obtained from Equations (14)–(17) as

(18) $$R^{\prime }=\frac{1-\frac{K_{0}\sigma_{s}}{E}}{1+\frac{2RK_{1}\sigma_{s}}{Et_{eq}}}R$$

The bending moment M on the section can be expressed as

(19) $$M=\int_{A}\sigma ydA=2\int_{0}^{\frac{t_{eq}}{2}}\sigma b_{p}ydy$$

Substituting (12) and (13) into (19) gives

(20) $$M={\;\sigma }_{s}\frac{b_{p}{t_{eq}}^{2}}{4}+\frac{E_{2}}{R^{\prime }}\frac{b_{p}{t_{eq}}^{3}}{12}$$

Let $\sigma_{0}$ be the maximum stress caused by the bending moment provided by the plate-bending machine

(21) $$\sigma_{0}=\frac{M\cdot y_{0}}{I_{z}}$$

After being equivalently transformed into a homogeneous plate, $y_{0}$ is $t_{eq}/2$, and $I_{z}$ is the section moment of inertia of the homogeneous plate.

Since the filler height is selected to be slightly higher than the rib top surface, the top of the rib only experiences the normal support force transmitted by the filler along the z-direction and the maximum compressive force along the x-direction caused by the bending moment. The stress is most concentrated, and since there is no skin constraint on the upper boundary, it becomes the most sensitive area to buckling.

**Figure 5:**
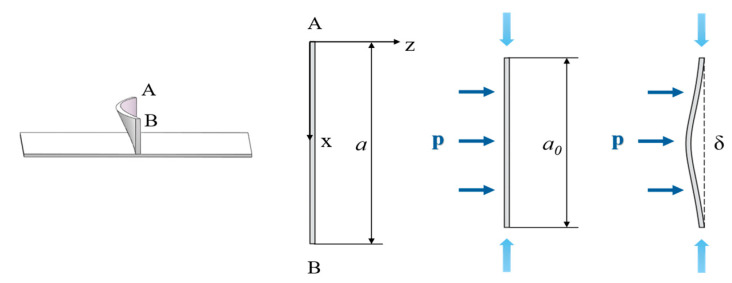
Rib buckling analysis model.

As shown in Figure 5, a section with a small thickness at the top of the rib is selected, assuming the section has a unit length of 1 along the rib height direction. This simplification reduces the problem to a two-dimensional plane, and the energy method is applied for analysis by analogizing the section to strut buckling with normal support.

The buckling mode equation for a strut with both ends fixed is $w\;=\;B\left(cos\frac{2n\pi x}{l}\;-\;1\right)\;$ [20], where n represents the number of buckling waves. As the axial load increases, the strut experiences instability, and the single-wave symmetric buckling, which requires the least energy, will occur first. Therefore, n = 1 is taken for analysis, assuming the buckling mode of the strut is

(22) $$w=\frac{\delta }{2}\left(cos\frac{2\pi x}{a_{0}}-1\right)$$

δ is the maximum instability height, and $a_{0}$ is the length after compression.

Let the total compression caused by the pressure provided by the bending moment be $u_{x}$. Assuming that no buckling deformation occurs after the rib is compressed, the engineering strain is

(23) $$\varepsilon_{eng}=\frac{\left(a-u_{x}\right)-a}{a}=-\frac{u_{x}}{a}$$

Based on the relationship between true strain and engineering strain $\varepsilon_{x}=ln(1\;+\;\varepsilon_{eng})$, it can be obtained that

(24) $$\varepsilon_{x}=-ln\left(1-\frac{u_{x}}{a}\right)$$

At this point, $\varepsilon_{x}$ is the ideal compression strain. For convenience in subsequent calculations, the compression strain is taken as a positive value, and it can further be obtained that

(25) $$a_{0}=ae^{-\varepsilon_{x}}$$

Based on the assumptions of incompressibility and constant thickness, the expression for the maximum compression instability wave height is derived using the arc length curve integral formula and Taylor expansion approximation. While these assumptions simplify calculations in plastic deformation, they neglect localized thickness increase in real metals under large strain. This may underestimate strain energy and rib buckling risk, particularly during small radius bending with high deformation, compromising model accuracy in these scenarios.

(26) $$a=\int_{0}^{a_{0}}\sqrt{1+{\;w^{\prime }}^{2}}dx\approx \int_{0}^{a_{0}}1+\frac{1}{2}{w^{\prime }}^{2}dx$$

(27) $$\delta =\frac{2}{\pi }\sqrt{u_{x}a_{0}}=\frac{2a}{\pi }\sqrt{e^{-\varepsilon_{x}}-e^{-2\varepsilon_{x}}}$$

A power-exponential function is used to describe the material’s strain-hardening behavior as it enters the plastic stage:

(28) $$\bar{\sigma }\;=\;K{\bar{\varepsilon }}^{n}$$

where K is the strength coefficient, and n is the strain-hardening exponent.

Under $\varepsilon_{y}\;=\;0$ plane strain conditions, it follows from the volume constancy condition that $\varepsilon_{z}\;=\;-\varepsilon_{x}$,

(29) $$\bar{\varepsilon }=\frac{\sqrt{2}}{3}\sqrt{{\left(-\varepsilon_{x}\right)}^{2}+{\left(\varepsilon_{x}-\varepsilon_{z}\right)}^{2}+{\left(\varepsilon_{z}\right)}^{2}}=\frac{2}{\sqrt{3}}\varepsilon_{x}$$

Based on the volume invariance before and after sectional compression,

(30) $$V=at_{st}$$

The plastic strain energy of the ideally compressed section is

(31) $$E_{0}=∭\bar{\sigma }d\bar{\varepsilon }dV=\frac{Kat_{st}}{n+1}{\left(\frac{2}{\sqrt{3}}\varepsilon_{x}\right)}^{n+1}$$

After compression instability, the bending strain energy of the section can be expressed as

(32) $$E_{w}=\iint \left(\int_{0}^{\varepsilon_{w}}\sigma_{w}d\varepsilon_{w}\right)drds$$

(33) $$\varepsilon_{w}=ln(r/r_{u})$$

$\varepsilon_{w}$ is the local strain due to bending, r is the radius of curvature at any point on the bending section, and $r_{u}$ is the radius of curvature at the neutral layer of the bending section.

(34) $$r_{u}=-\frac{{\left(1+{w_{0}^{\prime }}^{2}\right)}^{\frac{3}{2}}}{w_{0}^{\prime \prime }}+\frac{t_{st}}{2}=\frac{{a_{0}}^{2}}{2\pi^{2}\delta }+\frac{t_{st}}{2}$$

Further,

(35) $$E_{w}=\frac{2Kt_{st}}{n+1}{\left(\frac{t_{st}}{\sqrt{3}}\right)}^{n+1}{\left(\frac{2}{{\left(\frac{2\pi }{a_{0}}\right)}^{2}\delta }+\frac{t_{st}}{2}\right)}^{-n}arctan\left(\frac{\pi \delta }{a_{0}}\right)$$

According to the conservation of energy, write the energy equation.

(36) $$W_{p}={\;E}_{0\;}-E_{w}=\int_{0}^{\delta }Fdu_{z}$$

p is the normal supporting pressure brought by the filler.

The relationship between the external force F and the instability height $u_{z}$ is assumed to be [23].

(37) $$F=F_{max}-F_{max}{\left(\frac{u_{z}}{\delta }-1\right)}^{2}$$

Combining (36) and (37) yields

(38) $$p=\frac{3\left(E_{0}-E_{w}\right)}{2\delta a}$$

(39) $$p=\frac{3Kt_{st}}{2\delta \left(n+1\right)}\left(a{\left(\frac{2}{\sqrt{3}}\varepsilon_{x}\right)}^{n+1}-\frac{2}{a}{\left(\frac{t_{st}}{\sqrt{3}}\right)}^{n+1}{\left(\frac{2}{{\left(\frac{2\pi }{a_{0}}\right)}^{2}\delta }+\frac{t_{st}}{2}\right)}^{-n}arctan\left(\frac{\pi \delta }{a_{0}}\right)\right)$$

Assuming a is a multiple of the rib thickness, $a\;=\;\lambda t_{st}$, where λ is a normalized length and an arbitrary real number. Continuing the simplification yields [24]

(40) $$p=\frac{3K\pi {\left(e^{-\varepsilon_{x}}-e^{-2\varepsilon_{x}}\right)}^{-1/2}}{4\lambda \left(n+1\right)}\left({\left(\frac{2}{\sqrt{3}}\varepsilon_{x}\right)}^{n+1}-\frac{2}{\lambda 3^{(n+1)/2}}{\left(\frac{\lambda \sqrt{e^{3\varepsilon_{x}}-e^{2\varepsilon_{x}}}}{4\pi }+\frac{1}{2}\right)}^{-n}arctan\left(2\sqrt{e^{\varepsilon_{x}}-1}\right)\right)$$

**Figure 6:**
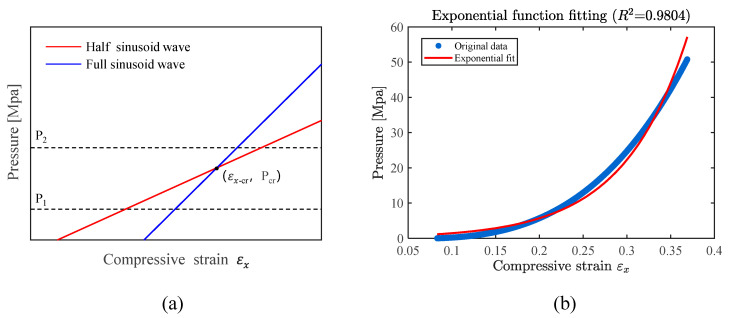
Pressure–compressive strain relationship: (**a**) functional relationships among different buckling modes, (**b**) exponential function fitting.

Research has shown that under different modes, there exist distinct functional relationships between normal pressure and compressive strain [23]. For instance, as shown in Figure 6a, both the half-sine-wave mode and the full-sine-wave mode have their own specific normal pressure–compressive strain functions, and the two curves intersect at a certain point. When the actual pressure is below this intersection pressure, the compressive strain associated with the half-wave mode is smaller, causing the sheet to buckle preferentially in a half-wave pattern. Conversely, when the actual pressure exceeds the intersection pressure, the compressive strain for the full-wave mode is smaller, and the sheet buckles preferentially in a full-wave pattern.

This implies that there exists a certain transition relationship between different modes. In this study, “modes” refer to half-waveforms of different wavelengths rather than changes in the waveform of the function itself. By establishing a theoretical model for the critical condition instability and solving for the intersection points of adjacent function curves, one can determine the minimum compressive strain at which instability occurs under that mode and the corresponding minimum supporting pressure. In order to establish a continuous relationship between supporting pressure and compressive strain, an increasing sequence of normalized length parameters (λ_1_, λ_2_, …, λₙ) is set. Based on

(41) $$p_{1}\left(\lambda_{k},\varepsilon_{x-cr}\right)=p_{2}\left(\lambda_{k+1},\varepsilon_{x-cr}\right)$$

use MATLAB 2023b to calculate the adjacent rib lengths, which correspond to the intersection compressive strains under different modes. Then, substitute these values back into Equation (40) to compute p for various values of $\varepsilon_{x}$. This helps construct the curve of supporting pressure versus compressive strain, and through curve fitting, derive its functional relationship, as shown in Figure 6b.

(42) $$p=f\;(\varepsilon_{x})$$

Use

(43) $$\sigma_{0}=\sigma_{s}+K{\left(\varepsilon_{x}\right)}^{n}$$

to obtain $\varepsilon_{x}$, and then obtain the corresponding pressure p.

**Figure 7:**
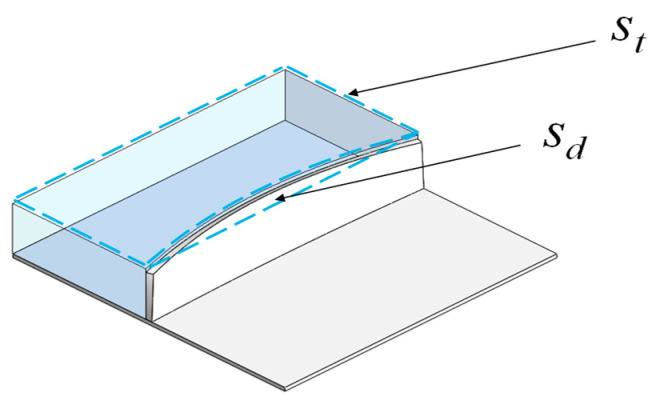
Filler deformation model.

The elastic modulus of the filler can be calculated using the following formula based on the stress–strain relationship, the specific deformation behavior is shown in Figure 7.

(44) $$E_{f}=\frac{\sigma }{\varepsilon }=\frac{p}{\frac{s_{d}}{s_{t}}}$$

where $s_{t}$ is the total cross-sectional area of the filler, and $s_{d}$ is the deformation area of the filler, which can be obtained by integrating Equation (22) from 0 to α.

After obtaining the elastic modulus $E_{f}$ of the filler, compare it with the initially assumed value $E_{1}$. If the error is within 10%, output $E_{f}$. If the error exceeds 10%, change the initial assumption $E_{1}$ to $E_{f}$ and rederive it, repeating until the condition is met and the accurate $E_{f}$ is output.

From the skin perspective, ensuring that the filler and the web bend together only requires that they have equal bending stiffness. Using a single grid cell as the study object, equating the bending stiffness of the skin and filler yields a filler elastic modulus that is far lower than the value derived from the rib buckling perspective. Therefore, the lower bound of the elastic modulus should be determined from the perspective of rib buckling.

In engineering practice, the principle of “just enough is best” is usually followed; a higher modulus is not always better. In fact, an excessively high modulus increases cost and technical difficulty, and since material hardness rises with elastic modulus, an overly hard filler can even cause the skin to punch through at the rib roots during the manufacture of high-rib thin-web grid-stiffened panels. Therefore, the elastic modulus of the filler should be selected based on the minimum value that meets forming requirements.

Applying the above theoretical calculation method to the grid-stiffened panel dimensions studied by Zhang’s team [19], the optimal elastic modulus of the filler was determined to be 1200 MPa by calculation. This value lies within the 450–1300 MPa range specified in their paper, demonstrating that the theoretical calculation method has good general applicability.

## 3. Simulation and Experiment

### 3.1. Simulation

**Figure 8:**
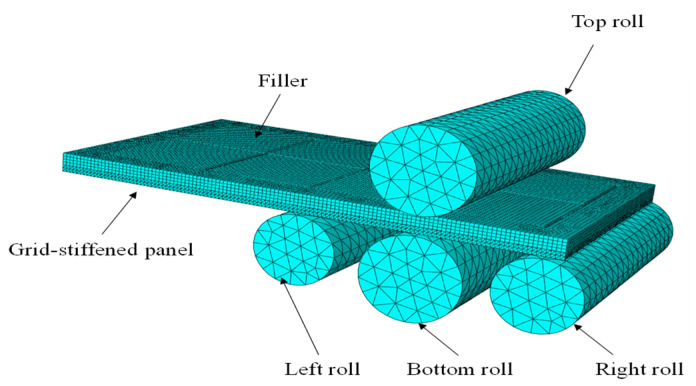
Four-roll simulation model.

As shown in Figure 8, a 3D simulation model of the aluminum alloy integral panel for filling roll-bending forming is established using ABAQUS 2023. The process material of the integral panel is 2A12-T4 aluminum alloy [19,25].

Table 1 shows the material properties of 2A12-T4 aluminum alloy.

Roll bending of sheet metal into a circular shape is a highly nonlinear process, and an explicit dynamic analysis was employed to avoid convergence difficulties. The simulation model includes two deformable bodies, the integral panel and the filler. The four rollers remain undeformed during the roll bending process and are defined as analytical rigid bodies. In terms of material, an isotropic, homogeneous elastoplastic model was adopted, with key parameters of the mesh panel introduced, including elastic modulus, mass density, Poisson’s ratio, and stress–strain relationship. For mesh discretization, the 3D model consists of a total of 215,252 nodes and 125,808 elements, among which the filler part is composed of 37,904 linear hexahedral elements with the element type being C3D8R, and the mesh panel part comprises 87,904 quadratic tetrahedral elements with the element type being C3D10M. For contact settings, general contact was used between the top and bottom rolls and the sheet metal, with contact properties defined by penalty function friction and a friction coefficient of 0.3; a tie constraint was applied between the filler and the panel.

The roll bending process is divided into three stages: clamping, bending, and unloading. In the clamping stage, the top roll is constrained in X and Y translational directions and rotations around all axes, with a Z-direction translation of −2; the right roll is constrained in X and Y translational directions and rotations around all axes, with a Z-direction translation of 20, achieving clamping and pre-bending of the sheet metal. In the bending stage, the top roll and the right roll are released from rotation around the Y-axis, the left roll has a Z-direction translation of 20, and all three rolls maintain constraints in X and Y translational directions and rotations around the X and Z axes; the bottom roll is constrained in all translational directions and rotations around the X and Z axes, and its rotation around the Y-axis changes from 7 radians to −10 radians, completing the reciprocating roll bending of the sheet metal. In the unloading stage, all rolls return to their initial positions along a prescribed path, bringing the entire roll-bending operation to an end.

To enhance computational efficiency, springback is omitted in the simulation for three reasons. First, it only marginally influences buckling displacement and generatrix straightness, which are this study’s primary metrics. Second, omitting springback still enables accurate validation of the theory and provides reliable guidance for experiments. Third, its exclusion significantly streamlines the model, reduces computation time, and greatly improves simulation efficiency.

Simulation analyses were conducted for roll-bending forming with curvature radii of 500 mm and 1675 mm. Based on the preceding theoretical calculations, the optimal filler elastic moduli were 1200 MPa and 1100 MPa, respectively. To systematically assess the effect of modulus variation, a ±500 MPa gradient was applied around each target value, yielding three cases per radius: 700, 1200, and 1700 MPa for the 500 mm radius, and 600, 1100, and 1600 MPa for the 1675 mm radius.

Continuous simulation adjustments to achieve the target curvature radius revealed that, with springback neglected and all process parameters held constant, varying filler elastic moduli produced different formed curvature radii in the grid-stiffened panels. The impact of the elastic modulus can be summarized in three key points. First, it affects stress transmission efficiency: high modulus fillers deform minimally under compression, efficiently transmitting roll pressure, while low modulus fillers disperse pressure, leading to insufficient stress transfer. Second, it influences contact conditions: high modulus fillers maintain stable contact, ensuring uniform pressure distribution and even bending, whereas low modulus fillers cause uneven pressure and curvature deviation. Third, it governs energy distribution: high modulus fillers absorb less energy in deformation, allowing more roll energy to convert into the panel’s plastic deformation, while low modulus fillers absorb more energy, reducing the energy applied to the panel and forcing an increase in curvature radius.

**Figure 9:**
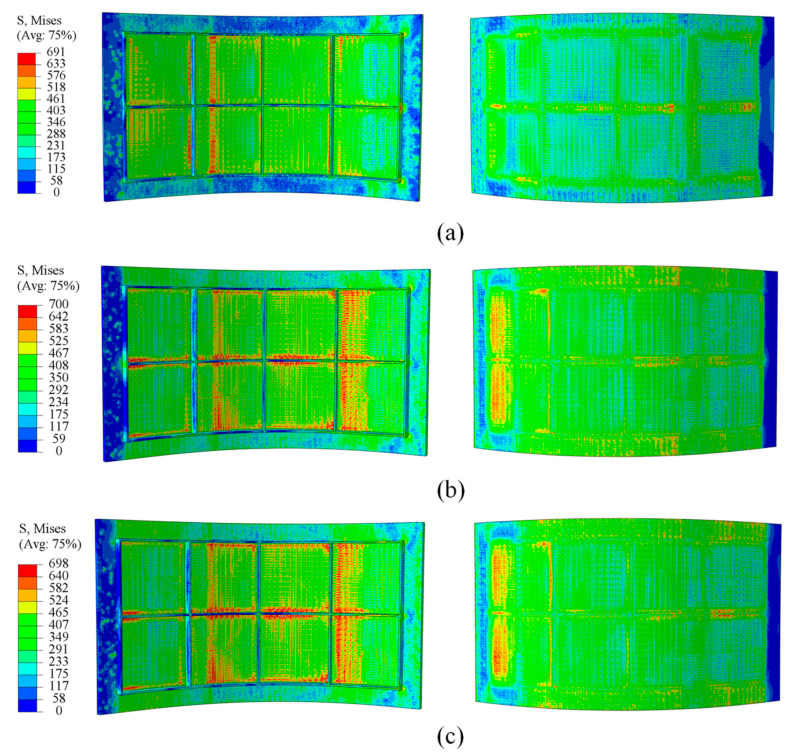
Stress distribution at a curvature radius of 500 mm under different filler elastic moduli: (**a**) 700 MPa; (**b**) 1200 MPa; (**c**) 1700 MPa.

**Figure 10:**
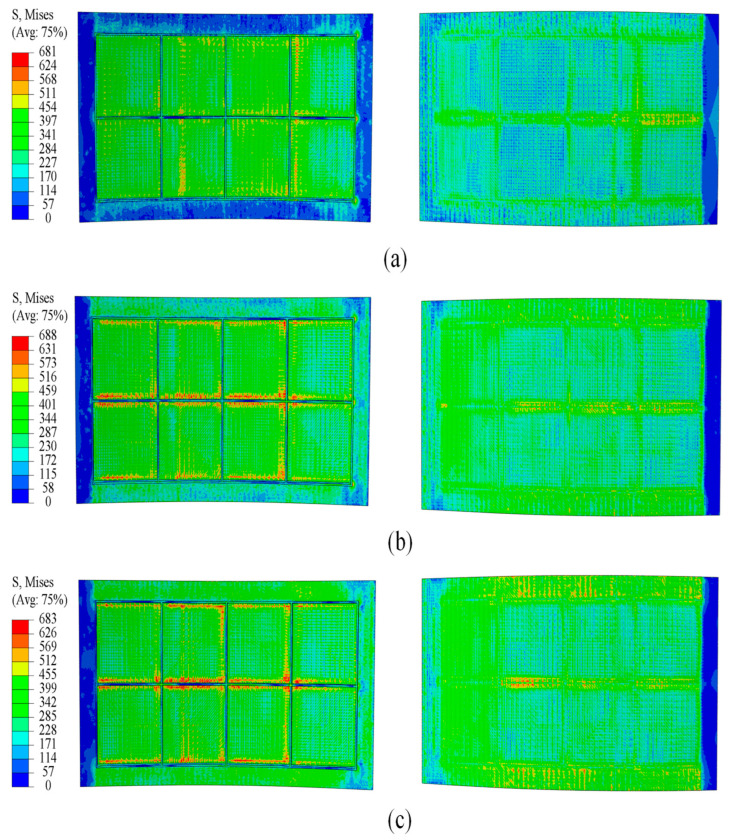
Stress distribution at a curvature radius of 1675 mm under different filler elastic moduli: (**a**) 600 MPa; (**b**) 1100 MPa; (**c**) 1600 MPa.

As shown in Figure 9 and Figure 10, a comparative analysis of simulation at two curvature radii indicates that fillers with elastic moduli of 1200 MPa/1700 MPa and 1100 MPa/1600 MPa produce a more uniform stress distribution than those with moduli of 700 MPa and 600 MPa. These higher modulus fillers effectively reduce the stress differential between the skin and ribs, ensuring more balanced loading across the panel. This stress distribution characteristic contributes to improved forming quality of the panel and a reduction in potential defects.

**Figure 11:**
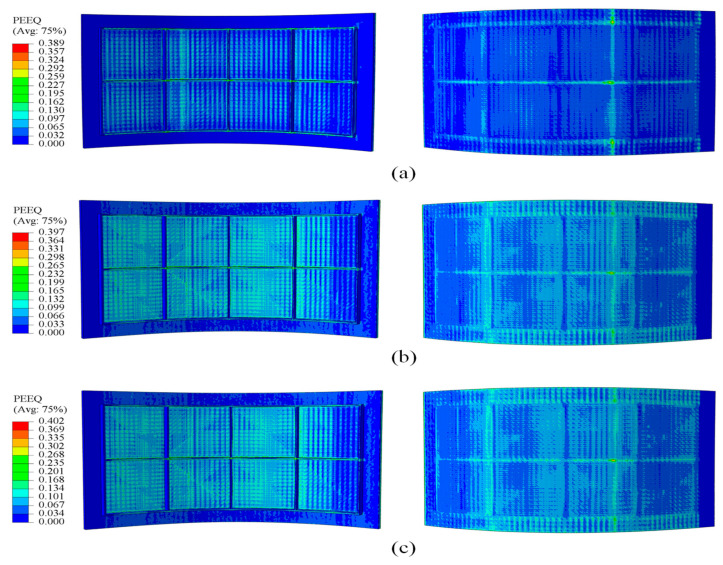
Strain distribution at a curvature radius of 500 mm under different filler elastic moduli: (**a**) 700 MPa; (**b**) 1200 MPa; (**c**) 1700 MPa.

**Figure 12:**
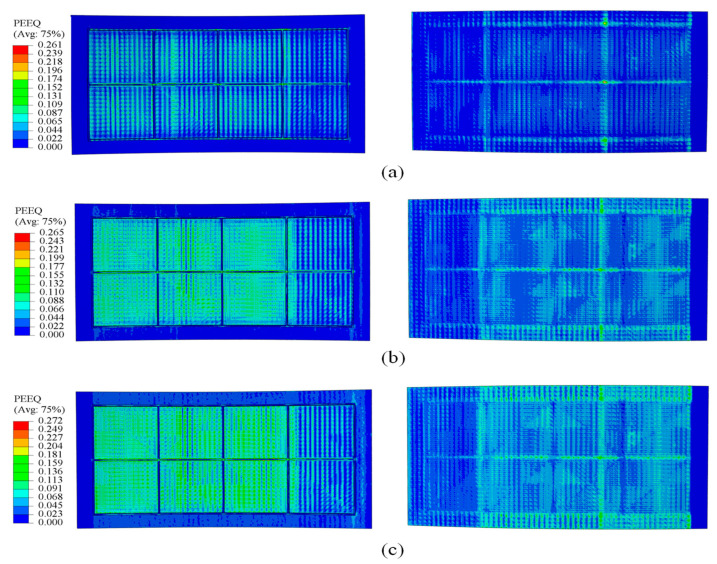
Strain distribution at a curvature radius of 1675 mm under different filler elastic moduli: (**a**) 600 MPa; (**b**) 1100 MPa; (**c**) 1600 MPa.

Figure 11 and Figure 12 show that, during forming, strain concentrates at the rib roots, particularly at the transverse ribs, where large deformations can induce material damage, impair the panel skin, and degrade rib-geometry accuracy. By increasing the elastic modulus of the filler material, the overall strain field becomes more uniform, peak strains at the rib roots are reduced, and deformation is more evenly distributed throughout the panel. This uniformity lowers the risk of crack initiation at the rib roots and helps preserve both skin integrity and rib-forming accuracy.

**Figure 13:**
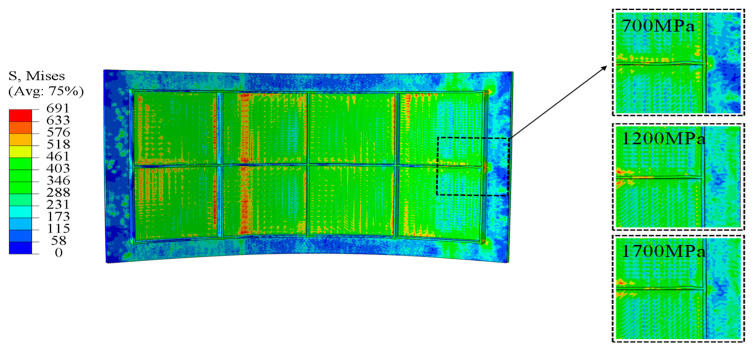
Buckling defect of rib.

At a curvature radius of 500 mm, panels filled with 700 MPa fillers displayed severe rib buckling as shown in Figure 13, resulting in pronounced forming defects. When the filler modulus is raised to 1200 MPa and 1700 MPa, forming defects at this curvature are substantially mitigated. These indicate that the higher modulus fillers maintain stable support stiffness throughout bending, effectively suppressing rib instability and markedly improving the panel’s overall forming quality.

During post-processing of the roll-bending simulation, application of an adjusted deformation scaling factor revealed a convex defect of the web plate. To mitigate this defect and preserve forming quality, appropriate process measures were used in the experiment, restraining its outward displacement in the thickness direction, and forming a bidirectional constraint in conjunction with the filler. Rounded fillets were introduced at rib roots and junctions to mitigate stress concentrations. A 1 mm gap was maintained between the filler and ribs to accommodate fillet geometry and ensure effective contact, thereby preventing interference and allowing coordinated material deformation during roll-bending forming.

### 3.2. Experiments

**Figure 14:**
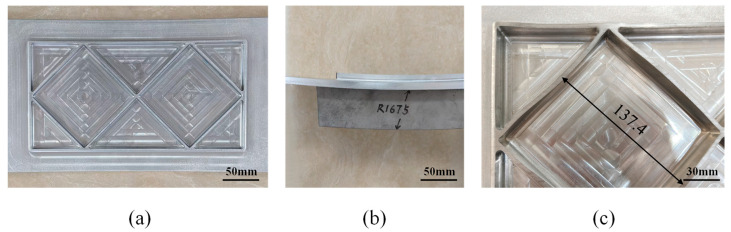
Test specimen: (**a**) inclined positioned orthogonal grid-stiffened panel, (**b**) radius of curvature measurement method, (**c**) buckling defect of ribs.

In the simulation, the filler effectively suppressed rib buckling under a curvature radius of 500 mm. To test the wider applicability of the elastic modulus calculation method, we employed an inclined orthogonal grid-stiffened panel shown in Figure 14a with a curvature radius of 1675 mm under suboptimal curvature conditions. Although the theoretical model focuses on rib buckling, generatrix straightness remains a critical quality metric, and because the inclined grid is more prone to generatrix straightness deviations during roll bending, this panel configuration was selected for experimental validation.

Four sets of inclined orthogonal grid-stiffened panels, all sharing identical geometry, were formed to a target curvature radius of 1675 mm: one group without filler and three groups with internal fillers having elastic moduli of 600 MPa (polytetrafluoroethylene), 1100 MPa (nylon 11), and 1600 MPa (nylon 11). All specimens were produced by milling, followed by filling roll bending. After forming, the fillers were removed, and forming quality was evaluated via caliper measurements and inspection of key parameters. As shown in Figure 14b, all three filled groups achieved the target radius, although their forming quality differed appreciably.

The empty-bending group exhibited the poorest performance as shown in Figure 14c, with all ribs experiencing severe buckling and the skin displaying pronounced inward concavity. This defect not only introduced substantial geometric deviations from the design intent but also critically compromised the panel’s load-bearing capacity, thus warranting classification as a failed specimen. In the absence of filler support, roll pressure acted directly upon the ribs, initiating buckling. Moreover, without the filler to facilitate load transfer, the skin was incapable of deforming as intended, resulting in a pronounced inward concavity.

The grid-stiffened panel incorporating a filler with an elastic modulus of 1600 MPa developed a convex defect on the underside of the web. The root cause lies in the filler’s elevated elastic modulus. When raised to 1600 MPa, the filler’s stiffness increases to the point that its capacity for compressive deformation is substantially reduced. During roll bending, this diminished deformability prevents the filler from coordinately distributing roller pressure across the web; the concentrated load leads to the characteristic convexity on the web’s underside.

## 4. Results and Discussion

**Figure 15:**
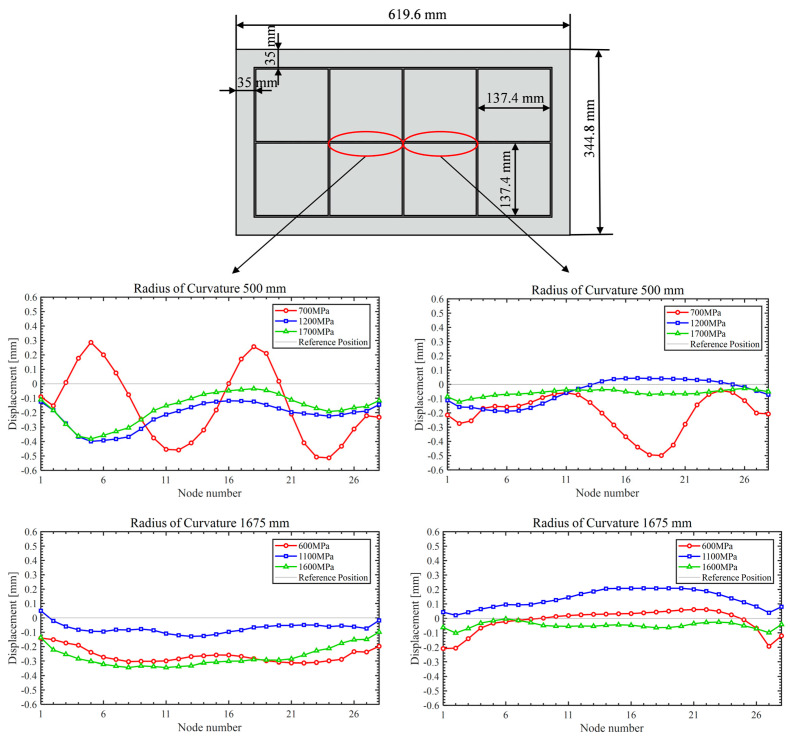
Rib buckling simulation results.

**Figure 16:**
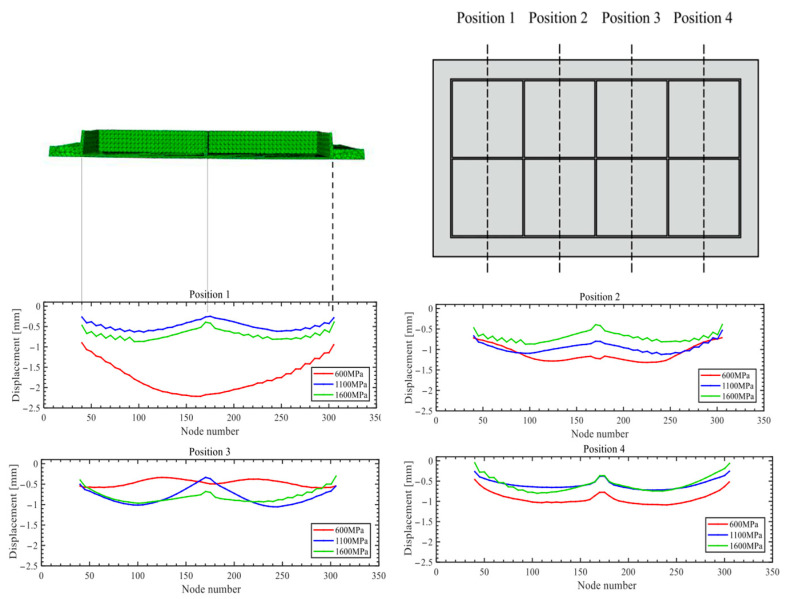
Generatrix straightness simulation results.

**Figure 17:**
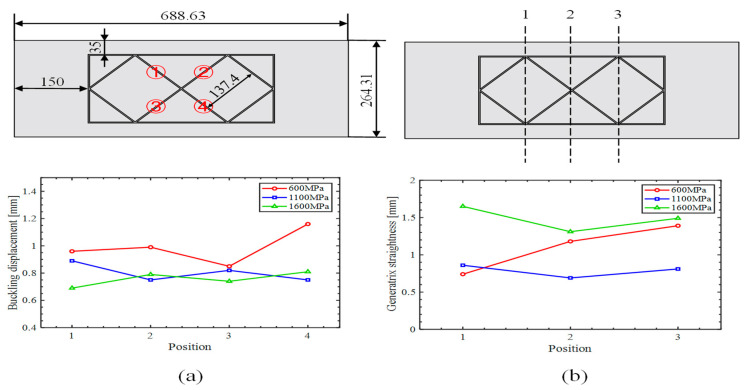
Experimental results: (**a**) rib buckling, (**b**) generatrix straightness.

Figure 15 presents the simulation results for rib buckling, while Figure 16 shows the simulation results for generatrix straightness, and Figure 17 displays the experimental results.

### 4.1. The Buckling Displacement of the Rib

Considering the simulation findings, two ribs at the midsection of the panel were chosen for measurement. The mid-panel region is minimally affected by the entry roll and does not require consideration of forming margin area parameters, thus yielding data that are more representative. Displacement values for rib nodes with curvature radii of 500 mm and 1675 mm were extracted separately and plotted as shown in Figure 15. At 500 mm radius, rib buckling displacement is strongly influenced by the filler’s elastic modulus. Specifically, the 700 MPa group exhibits a peak displacement of 0.50 mm, whereas the 1700 MPa group shows the smallest peak displacement of 0.35 mm. This result demonstrates that higher modulus fillers significantly suppress rib buckling at small curvature radii. At a large curvature radius of 1675 mm, the modulus of elasticity has little effect in inhibiting rib buckling. However, the minimum node displacement occurs at the theoretically calculated optimal modulus of 1100 MPa.

Figure 17 presents the measured central-rib buckling displacements of inclined orthogonal grid-stiffened panels. At elastic moduli of 600, 1100, and 1600 MPa, the average displacements were 0.99 mm, 0.81 mm, and 0.76 mm, respectively. These experimental results confirm that increasing the filler’s modulus of elasticity effectively reduces rib buckling displacement, in excellent agreement with our theoretical analysis.

In summary, both simulations and experiments demonstrate a consistent trend: increasing the filler’s elastic modulus enhances rib buckling resistance. This effect is particularly pronounced in simulations for panels with small curvature radii, where selecting a higher modulus filler significantly suppresses rib buckling and tightly controls node displacement.

### 4.2. Generatrix Straightness

Based on the simulation results, the generatrix straightness at a curvature radius of 1675 mm was measured at four key central grid points, and the web’s deformation characteristics are vividly illustrated using visualized plots as shown in Figure 16. Overall, the web exhibits a convex deformation trend across all elastic moduli tested. However, in the 600 MPa case, positions 2 and 3 display a concave deflection, indicating that such a low modulus cannot effectively suppress skin indentation. Generatrix straightness decreases as modulus increases. The average straightness values are 0.765 mm for 600 MPa, 0.725 mm for 1600 MPa, and 0.510 mm for 1100 MPa. Notably, the 1100 MPa group outperforms the 600 MPa and 1600 MPa cases by 33.4% and 29.7%, respectively.

Measurements were taken at the three positions shown in Figure 17, using a straightedge applied to the panel exterior, and the maximum gap at each position was recorded to characterize generatrix straightness. The measured generatrix straightness values under elastic modulus settings of 600 MPa, 1100 MPa, and 1600 MPa are 1.48 mm, 0.82 mm, and 1.06 mm, respectively. These data indicate that appropriate process measures can effectively mitigate skin convexity but have only a limited effect on concave defects. Notably, the specimen processed with a 1100 MPa elastic modulus exhibited the lowest average gap of 0.82 mm, corresponding to the best forming quality. These findings validate the suitability of the inclined orthogonal grid and confirm that selecting an optimal elastic modulus can significantly improve generatrix straightness in roll-bending forming.

Both simulation and experimental results consistently demonstrate that the optimal elastic modulus yields the best generatrix straightness. While simulations showed the 1100 MPa modulus outperforming the 1600 MPa case by a significant 29.7%, the experimental advantage was narrower at 22.6%. This reduced margin in experiments is attributed to the application of certain constraints on the web plate for both specimens. Since the 1600 MPa specimen had more severe initial outward convexity, the constraints brought a greater improvement, narrowing the gap.

**Figure 18:**
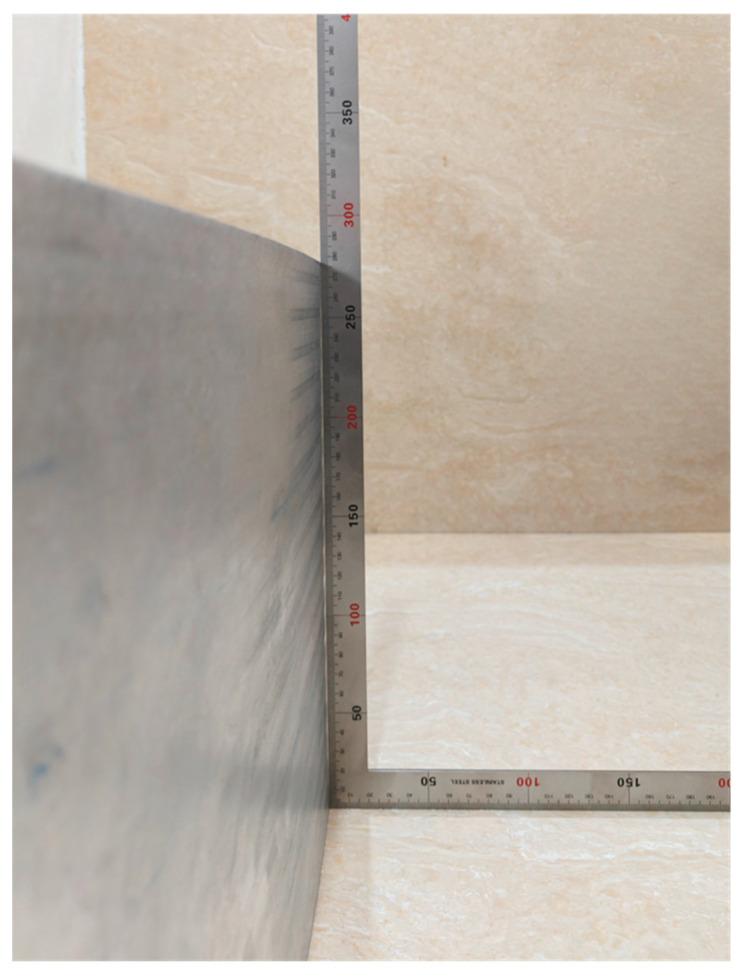
Generatrix straightness measurement method.

Figure 18 shows the generatrix straightness measurement method.

## 5. Conclusions

This study systematically addresses rib buckling and skin deformation defects in high-rib thin-web grid-stiffened panels during roll bending by establishing a methodology for optimizing filler elastic modulus. Key findings and implications are summarized as follows:

1. Validated Theoretical Framework

A computational method for filler elastic modulus was developed based on rib-buckling theory and stiffness equivalence principles, establishing a direct link between modulus and critical buckling load. The theoretically derived optimal moduli effectively suppressed rib buckling in simulations and experiments. Experimental results confirmed the model’s applicability: filler moduli of 1100 MPa and 1600 MPa significantly reduced buckling compared to 600 MPa, and the 1100 MPa filler produced the best generatrix straightness.

2. Critical Role of Filler Elastic Modulus

Low modulus leads to inadequate support, causing severe rib buckling and skin concavity. Fillers within the optimal elastic modulus enable efficient stress transmission and coordinated deformation, significantly reducing rib buckling displacement while promoting uniform stress and strain distribution, substantially improving generatrix straightness. Excessive modulus causes diminished compressive compliance, leading to local overloading and convex defects on the web underside.

3. Engineering Application Potential

The methodology provides a direct theoretical basis for selecting filler properties to prevent rib buckling in high-precision roll bending, moving beyond trial-and-error approaches. Combining the optimal elastic modulus with appropriate processing techniques effectively mitigates key defects like rib buckling and skin concavity/convexity, enhancing forming accuracy for critical aerospace components.

4. Future Research Directions

Future studies should prioritize simplifying theoretical calculations by developing weight-based, concise formulas suitable for direct engineering application, extending the methodology to mainstream non-orthogonal grid types such as triangular configurations through systematic validation, and exploring the upper-bound limit of the elastic modulus from the skin-coordination perspective to complement the existing rib-buckling-derived optimization criterion, while further refining bidirectional constraint techniques to manage web convexity.

## Figures and Tables

**Table 1:** Material properties of 2A12-T4 aluminum alloy.

Material	Elastic Modulus, *E*/MPa	Initial Yield Stress, $\sigma_{S}$/MPa	Poisson Ratio, ν	Mass Density, kg/m^3^	Strengthening Coefficient, *K*/MPa	Strain-Hardening Exponent, *n*
aluminum alloy 2A12-T4	72,400	325	0.33	2770	989	0.654

## Data Availability

The data presented in this study are available on request from the corresponding author. The data are not publicly available due to privacy restrictions.

## Nomenclature

a	Length of the strut before compression $\left[mm\right]$
$a_{0}$	Length of the strut after compression $\left[mm\right]$
$a_{m}$	Length of the margin area $\left[mm\right]$
b	Spacing between ribs $\left[mm\right]$
$b_{p}$	Width of the plate $\left[mm\right]$
B	Bending stiffness of a single rib $\left[N\cdot m^{2}\right]$
$D_{x}$	Effective bending stiffness in the x direction $\left[N\cdot m\right]$
$E_{0}$	Plastic strain energy $\left[N\cdot m\right]$
$E_{1}$	Assumed elastic modulus of the filler $\left[MPa\right]$
$E_{2}$	Material’s linear hardening modulus $\left[MPa\right]$
$E_{f}$	The elastic modulus of the filler $\left[MPa\right]$
$E_{w}$	Bending strain energy $\left[N\cdot m\right]$
h	Height of the rib $\left[mm\right]$
$h_{m}$	Height of the margin area $\left[mm\right]$
K	Strength coefficient $\left[MPa\right]$
$K_{0}$	Relative hardening modulus $\left[-\right]$
$K_{1}$	Shape factor $\left[-\right]$
M	Bending moment on the section $\left[N\cdot m\right]$
n	Strain-hardening exponent $\left[-\right]$
p	Normal pressure $\left[MPa\right]$
r	Relative curvature radius after springback $\left[-\right]$
$r^{\prime }$	Relative curvature radius before springback $\left[-\right]$
R	Curvature radius after springback $\left[mm\right]$
$R^{\prime }$	Curvature radius before springback $\left[mm\right]$
$s_{d}$	Deformation area of the filler $\left[{mm}^{2}\right]$
$s_{t}$	Total cross-sectional area of the filler $\left[{mm}^{2}\right]$
$t_{eq}$	Equivalent thickness of the total homogeneous panel $\left[mm\right]$
$t_{f}$	Equivalent thickness of the filler $\left[mm\right]$
$t_{m}$	Equivalent thickness of the forming margin area $\left[mm\right]$
$t_{r}$	Equivalent thickness of the grid rib portion $\left[mm\right]$
$t_{sk}$	Thickness of the original skin $\left[mm\right]$
$t_{st}$	Thickness of the rib $\left[mm\right]$
$u_{x}$	Total compression $\left[mm\right]$
$u_{z}$	Instability height $\left[mm\right]$
w	Deflection $\left[mm\right]$
W	Section modulus $\left[m^{3}\right]$
$\sigma_{0}$	Maximum stress $\left[MPa\right]$
$\sigma_{s}$	Initial yield stress $\left[MPa\right]$
$\varepsilon_{x}$	Ideal compression strain $\left[-\right]$
